# Design of a high voltage gain converter using coupled inductor with reduced voltage stress for photovoltaic energy based systems

**DOI:** 10.1038/s41598-024-72600-y

**Published:** 2024-09-13

**Authors:** Seyed Majid Hashemzadeh, Seyed Hossein Hosseini

**Affiliations:** 1 Faculty of Electrical and Computer Engineering, University of Tabriz, Tabriz, Iran; 2 Engineering Faculty, Near East University, Mersin 10, 99138 Nicosia, North Cyprus Turkey

**Keywords:** DC-DC converter, High voltage gain, Coupled inductor, Voltage multiplier cells, Low voltage stress, Renewable energy, Electrical and electronic engineering, Energy infrastructure

## Abstract

This paper presents the design and analysis of a high voltage gain converter utilizing a coupled inductor with reduced voltage stress, specifically for photovoltaic energy-based systems. The proposed converter employs a two-winding coupled inductor and voltage multiplier cells to achieve an increase in output voltage while mitigating voltage stress across semiconductor components. Additionally, the voltage multiplier cells function as voltage clamps for the power switch, further enhancing the converter's performance. The converter features a single switch design, which simplifies control, reduces cost, and improves reliability. Key advantages of the converter include a low component count, a common ground between input and output ports, and high efficiency. The converter's performance is thoroughly investigated through mode analysis and steady-state analysis. Comparative evaluations with similar converters are conducted to highlight the benefits and performance of the proposed design. To validate the theoretical analysis, a 125 W prototype with 26 V input and 200 V output voltages operating at a 50 kHz switching frequency is developed, and experimental results are presented, demonstrating the effectiveness and practicality of the proposed high voltage gain converter.

## Introduction

The global shift towards clean and sustainable energy sources has catalyzed the advancement of renewable energy technologies^1,2^. Among these, photovoltaic (PV) systems stand out due to their ability to directly convert sunlight into electricity using semiconductor materials^3,4^. As a prominent and eco-friendly alternative to fossil fuels, PV systems are integral to reducing greenhouse gas emissions and achieving energy independence^5,6^. However, the efficiency and effectiveness of PV systems in practical applications hinge on advanced power conversion technologies that can manage the inherent characteristics of solar energy, such as variable output voltage and relatively low voltage levels^7,8^.

**Fig. 1:**
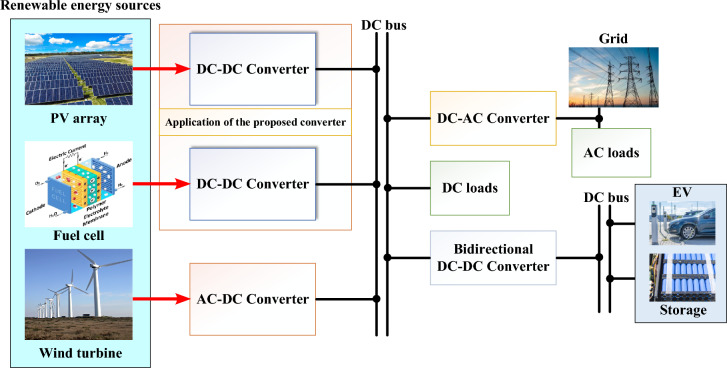
Power Electronics based power system.

Figure 1 shows the power electronics-based power system with integration of the renewable energy resources. A key technology in this domain is the high step-up DC-DC converter, which is essential for integrating PV systems into the electrical grid or standalone power systems. These converters are designed to boost the low voltage produced by PV panels to higher levels suitable for various applications, ensuring efficient power transfer, system stability, and compatibility with downstream components. PV panels typically generate a DC voltage that ranges between 18 and 45 V for individual modules^9^. For effective use in grid-tied inverters, battery charging systems, or direct load applications, this voltage needs to be increased significantly, to 200 V or higher. High step-up DC-DC converters are specifically designed to perform this voltage transformation efficiently, enabling the integration of PV systems with standard electrical infrastructure. Maximizing energy conversion efficiency is crucial for PV systems to make the most of the harvested solar energy^10,11^. High step-up DC-DC converters are engineered to minimize energy losses during the conversion process. Advanced designs and control techniques ensure that the converters operate at high efficiency across a wide range of operating conditions, thereby enhancing the overall performance and energy yield of PV systems^12,13^. Solar irradiance and temperature fluctuations can cause significant variations in the output of PV panels. High step-up DC-DC converters are equipped with sophisticated control mechanisms, including maximum power point tracking (MPPT) algorithms, which dynamically adjust the operating point of the PV panels to extract maximum power^14^. Additionally, these converters provide voltage regulation, ensuring a stable and consistent output voltage despite varying input conditions. Effective voltage boosting enables the use of standard inverters and other downstream components, reducing the overall system cost^15^. The high efficiency of modern converters translates into lower energy losses and improved system longevity, which in turn reduces operational and maintenance costs^16^. This cost-effectiveness is crucial for the widespread adoption of PV systems, especially in large-scale deployments and residential applications^17^. Several topologies are employed in the design of high step-up DC-DC converters, each offering distinct advantages tailored to different PV system requirements such as:

(1) Boost Converter: This basic topology uses an inductor, switch, diode, and capacitor to increase the input voltage. It is simple and cost-effective but may not achieve extremely high voltage gains without compromising efficiency. Its straightforward design makes it suitable for low to moderate power applications^18^. (2) Flyback Converter: Combining the functions of a transformer and a boost converter, the flyback converter provides electrical isolation and higher voltage gain. It is suitable for applications requiring moderate power levels and isolation but may encounter issues with high voltage stress on components. (3) Push–Pull Converter: Utilizing two switches to drive a transformer, this topology offers higher efficiency and better performance at medium to high power levels^19^. It is favored for applications that demand robust performance and reliable operation. (4) Interleaved Boost Converter: By using multiple boost converters in parallel, this topology reduces input current ripple and improves overall efficiency. The interleaved approach enhances thermal performance and scalability, making it ideal for high power applications. (5) Cascade Boost Converter: Featuring multiple boost stages in series, this converter achieves very high voltage gain^20^. It is effective for applications that require significant voltage step-up while managing component stress and ensuring efficient operation. (6) Switched-Capacitor Converter: This topology uses capacitors as energy storage elements to achieve voltage boosting. It is highly efficient and suitable for integrated circuits and low-power applications where space and efficiency are critical. (7) Resonant Converters: These converters operate at resonant frequency to achieve high efficiency and reduced electromagnetic interference. They are suitable for applications requiring high power density and efficient thermal management^21^. (8) Coupled Inductor-Based Converters: These converters use coupled inductors to achieve higher voltage gain and improve efficiency. By sharing magnetic components, coupled inductor-based converters reduce size and losses associated with magnetic elements. This topology is advantageous for high power applications requiring significant voltage boost with improved efficiency and reduced electromagnetic interference^22^.

High step-up DC-DC converters are pivotal in various renewable energy applications beyond PV systems, including wind energy, fuel cells, and energy storage systems^23^. Their ability to provide efficient and reliable voltage transformation is crucial for the seamless integration of renewable energy sources into the existing power grid and emerging smart grid technologies. The future of high step-up DC-DC converters lies in the development of advanced materials, such as wide bandgap semiconductors (e.g., silicon carbide and gallium nitride), which offer superior efficiency and thermal performance^24^. Furthermore, advancements in digital control techniques and artificial intelligence (AI) can enhance the adaptability and performance of these converters, enabling real-time optimization and predictive maintenance. Generally, high step-up DC-DC converters are indispensable in modern PV systems, addressing the challenges posed by low and variable output voltages^25,26^. By efficiently boosting voltage levels, these converters enhance the viability and performance of PV systems, facilitating the broader adoption of renewable energy sources. Continued innovation in converter topologies, materials, and control strategies will further improve their efficiency, reliability, and cost-effectiveness, driving the growth of solar energy and other renewable applications worldwide^27^.

In the field of photovoltaic energy-based systems, achieving high voltage gain while minimizing voltage stress on semiconductor components is a critical challenge. This paper addresses this issue by presenting a novel high voltage gain converter that employs a coupled inductor with reduced voltage stress. The proposed converter incorporates a two-winding coupled inductor and voltage multiplier cells, not only to boost the output voltage but also to act as voltage clamps for the power switch, thereby enhancing overall performance. The design is streamlined with a single switch, simplifying control, reducing costs, and improving reliability. Key benefits of this converter include a low component count, a common ground between input and output ports, and high efficiency. A comprehensive investigation of the converter's function is conducted through mode analysis and steady-state analysis. Comparative studies with similar converters are provided to underscore the advantages and performance of the proposed design. To validate the theoretical analysis, experimental results are demonstrated to prove the effectiveness and practicality of the proposed high voltage gain converter.

## Operation modes analysis

**Fig. 2:**
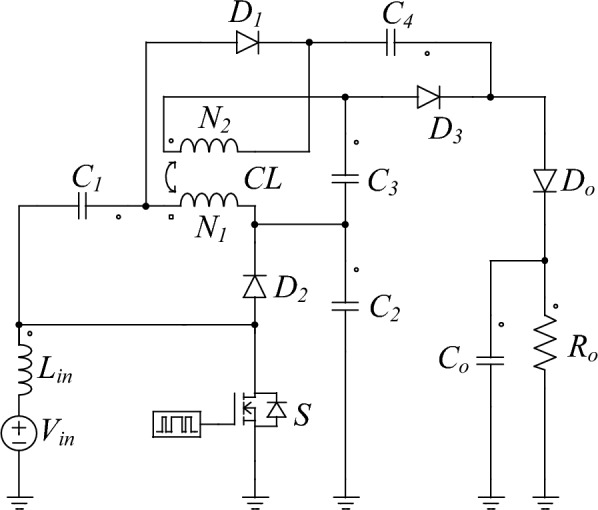
The presented DC-DC Converter.

The proposed DC-DC converter topology is shown in Fig. 2. This converter is designed to efficiently convert energy using a combination of inductors, capacitors, diodes, and switches. The primary components include: One power switch (*S*), four diodes (*D*_*1*_, *D*_*2*_, *D*_*3*_, *D*_*o*_), five capacitors (*C*_*1*_, *C*_*2*_, *C*_*3*_, *C*_*4*_, *C*_*o*_), One input inductor (*L*_*in*_), One coupled inductor with a turns ratio defined as *N* = *N*_*2*_*/N*_*1*_.

**Fig. 3:**
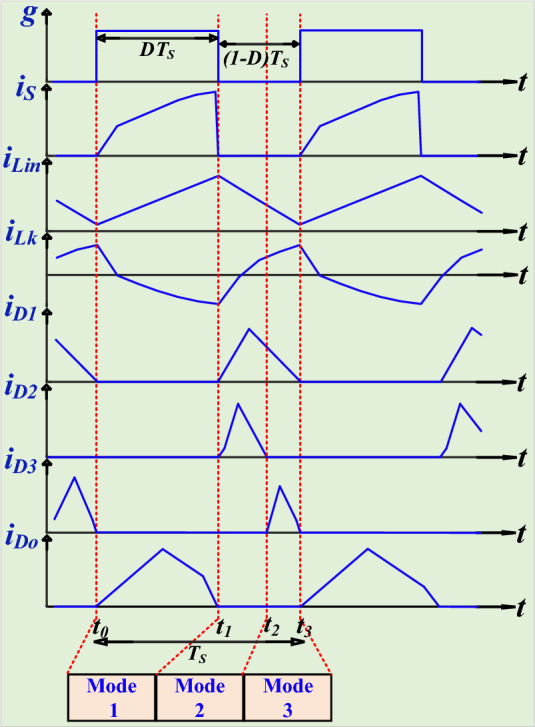
The key steady-state waveforms of the presented topology {R1-3}.

**Fig. 4:**
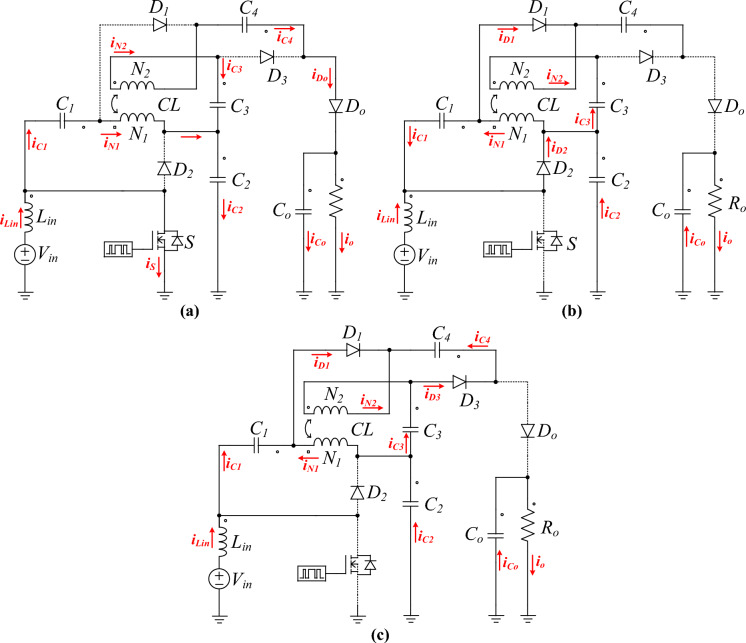
The operation modes in each switching period, (**a**) 1, (**b**) 2, and (**c**).

This configuration enables the converter to operate through different modes within each switching period, optimizing energy transfer and ensuring high efficiency. The main waveforms are depicted in Fig. 3, and equivalent circuit of each mode is visible in Fig. 4 (a)-(c).

**Mode 1 (*t***_***0***_** < *t* < *t***_***1***_**):** In this initial mode, the power switch S is turned on, allowing the inductor Lin to store energy from the input voltage source *V*_*in*_. During this phase, diodes *D*_*1*_, *D*_*2*_, and *D*_*3*_ are reverse-biased, while diode *D*_*o*_ is forward-biased, allowing current to flow through it. The current through the *L*_*in*_ increases linearly due to the constant voltage applied across it. Capacitors *C*_*1*_, *C*_*2*_, and *C*_*3*_ help in smoothing the voltage and reducing ripple by storing and releasing energy as needed. This mode is crucial for building up the energy in the inductor which will be transferred to the output port. Therefore, the energy stored in the magnetic field of the inductor is fully transferred to the load and output capacitors.

1 $$ V_{{L_{in} }} = V_{in} $$

2 $$ V_{{N_{1} }} = V_{{L_{m} }} = V_{{C_{1} }} - V_{{C_{2} }} $$

3 $$ V_{o} = V_{{C_{4} }} + V_{{C_{3} }} - NV_{{C_{1} }} - V_{{C_{2} }} + NV_{{C_{2} }} $$

4 $$ i_{in} = - i_{{C_{2} }} + i_{S} $$

5 $$ i_{{L_{k} }} = - i_{{C_{2} }} $$

6 $$ i_{{D_{o} }} = i_{{C_{o} }} + i_{o} $$

7 $$ i_{{C_{3} }} = i_{{C_{4} }} = - i_{{N_{2} }} = - i_{{D_{o} }} $$

**Mode 2 (*t***_***1***_** <  < *t* < *t***_***2***_**):** At the beginning of Mode 2, the power switch *S* is turned off. The energy stored in *L*_*in*_ is now released. Diode *D*_*o*_ becomes reverse-biased, and *D*_*1*_ becomes forward-biased, allowing current to flow through *D*_*1*_. This mode is characterized by the transfer of energy from the inductor *L*_*in*_ to the VMCs capacitors. The current through the inductor *L*_*in*_ decreases as energy is delivered to the load by output capacitors *C*_*o*_, while the voltage across the inductor reverses, driving the current through *D*_*1*_.

8 $$ V_{{L_{in} }} = V_{in} - V_{{C_{2} }} $$

9 $$ V_{{N_{1} }} = V_{{L_{m} }} = V_{{C_{1} }} $$

10 $$ V_{{C_{3} }} = V_{{L_{m} }} + NV_{{L_{m} }} $$

11 $$ i_{in} = i_{{C_{2} }} + i_{{D_{1} }} $$

12 $$ i_{{C_{3} }} = - i_{{N_{2} }} = i_{{D_{2} }} $$

**Mode 3 (*t***_***2***_** < *t* < *t***_***3***_**):** In Mode 3, the power switch *S* remains off, and diode *D*_*1*_ continues to conduct. This mode ensures the continuous delivery of energy to the load even when the power switch is off, providing a path for the inductor current to flow. The inductor *L*_*in*_ might have a residual current flowing through it, which gradually decreases. Diode *D*_*2*_ is in a blocking state, ensuring no reverse current flows back to the source, thus maintaining efficient operation. This mode helps to prevent oscillations and ensures smooth current transition before the cycle restarts with Mode 1.

13 $$ V_{{C_{4} }} = NV_{{L_{m} }} $$

14 $$ i_{{C_{4} }} = i_{{D_{3} }} $$

15 $$ i_{{C_{o} }} = - i_{o} $$

In the proposed DC-DC converter, the leakage energy of the coupled inductor is effectively managed and recycled to enhance the overall efficiency. During Mode 1, energy is stored in the leakage inductance. In Mode 2, this leakage energy is transferred to capacitor *C*_*3*_ via diode *D*_*2*_, thereby reducing energy losses associated with leakage inductance. In Mode 3, any remaining leakage energy is fully transferred to the output, ensuring that no significant energy is wasted. The selection of components in the DC-DC converter is critical to ensure efficient operation across all modes. The power switch *S* must be capable of handling the peak current and voltage stresses without significant losses. Similarly, the diodes *D*_*1*_ through *D*_*o*_ should have low forward voltage drop and fast recovery times to minimize losses and ensure quick switching. Capacitors *C*_*1*_ through *C*_*o*_ need to have low Equivalent Series Resistance (ESR) to handle ripple currents efficiently. The inductor Lin plays a key role in energy storage and transfer. Its inductance value should be chosen to ensure continuous conduction mode (CCM) operation under normal load conditions, which helps in reducing ripple and improving the overall efficiency of the converter. The coupled inductor with its turns ratio *N* helps in adjusting the voltage levels and improving the power transfer capability of the converter. The proposed DC-DC converter efficiently manages energy transfer through these three operations modes. By appropriately switching the power switch and diodes, the converter ensures minimal energy loss and optimal performance.

## Anaylsis of steady-state operation

The proposed DC-DC converter's steady-state operation is characterized by the voltages across its components during each switching period. The key voltage relationships are derived based on the applied volt-second balance principle on the inductors and capacitors.

### Voltages

Applying volt-second balance principle over inductor *L*_*in*_ and magnetizing inductance of the coupled inductor (*L*_*m*_), the voltage of *C*_*1*_ and *C*_*2*_ are calculated versus input voltage:

16 $$ \left\langle {V_{{L_{in} }} } \right\rangle_{Ts} = 0\quad \to \quad V_{{C_{2} }} = \frac{1}{1 - d}V_{in} $$

17 $$ \left\langle {V_{{L_{m} }} } \right\rangle_{Ts} = 0\quad \to \quad V_{{C_{1} }} = dV_{{C_{2} }} = \frac{d}{1 - d}V_{in} $$

Using (10) and (13), the voltage over *C*_*3*_ and *C*_*4*_ can be determined:

18 $$ V_{{C_{3} }} = \frac{Nd + d}{{1 - d}}V_{in} $$

19 $$ V_{{C_{4} }} = \frac{Nd}{{1 - d}} $$

By replacing (16), (17), (18), and (19) into (3), the output voltage, and therefore the voltage gain are calculated as (2) and (21).

20 $$ V_{o} = \frac{Nd}{{1 - d}}V_{in} + \frac{Nd + d}{{1 - d}}V_{in} - \frac{Nd}{{1 - d}}V_{in} + \frac{1 + N}{{1 - d}}V_{in} $$

21 $$ M = \frac{{V_{o} }}{{V_{in} }} = \frac{{1 + N + d\left( {1 + N} \right)}}{1 - d} $$

**Fig. 5:**
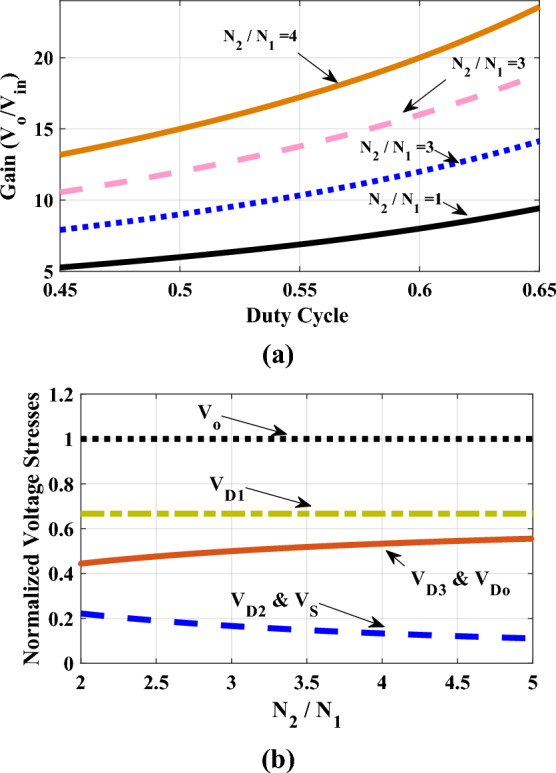
(**a**) voltage conversion versus D and *N*, (**b**) comparison of the normalized voltage stress.

Considering the obtained equations for capacitors and output port, the voltage stress across semiconductors can be obtained. The calculated equations for power switch and diodes voltage stresses are summarized at Table 1. Figure 5 (a) and (b) depict the voltage gain and normalized voltage stresses versus duty cycle and turns ratio of the coupled inductor.

**Table 1:** Voltage stress analysis.

Semiconductors	Voltage stress	Normalized voltage stress
*S*	$V_{S} = V_{{C_{2} }} = \frac{1}{1 - d}V_{in}$	$\frac{{V_{S} }}{{V_{o} }} = \frac{1}{{1 + N + d\left( {1 + N} \right)}}$
*D*_*1*_	$V_{{D_{1} }} = V_{{C_{3} }} - V_{{L_{m} }} - NV_{{L_{m} }} = \frac{1 + N}{{1 - d}}V_{in}$	$\frac{{V_{{D_{1} }} }}{{V_{o} }} = \frac{1}{1 + d}$
*D*_*2*_	$V_{{D_{2} }} = V_{{C_{2} }} = \frac{1}{1 - d}V_{in}$	$\frac{{V_{{D_{2} }} }}{{V_{o} }} = \frac{1}{{1 + N + d\left( {1 + N} \right)}}$
*D*_*3*_	$V_{{D_{3} }} = V_{{C_{4} }} - NV_{{L_{m} }} = \frac{N}{1 - d}V_{in}$	$\frac{{V_{{D_{3} }} }}{{V_{o} }} = \frac{N}{{1 + N + d\left( {1 + N} \right)}}$
*D*_*o*_	$V_{{D_{o} }} = NV_{{C_{1} }} - V_{{C_{2} }} - V_{{C_{3} }} - V_{{C_{4} }} + V_{o} = \frac{N}{1 - d}V_{in}$	$\frac{{V_{{D_{o} }} }}{{V_{o} }} = \frac{N}{{1 + N + d\left( {1 + N} \right)}}$

### Currents

Based on the configuration of the proposed topology, the average current of diodes *D*_*1*_, *D*_*3*_, and *D*_*o*_ are equal to the output current Io. Also, the input average current can be determined using voltage gain relation (*M* = *V*_*o*_*/V*_*in*_).

22 $$ I_{in} = MI_{o} = \frac{{1 + N + d\left( {1 + N} \right)}}{1 - d}I_{o} $$

Using KCL law, the average current of the power switch is calculated versus input and diode *D*_*1*_ average currents. The average current of *L*_*m*_ is also achieved versus input and power switch average currents.

23 $$ I_{S} = I_{in} - I_{{D_{1} }} = \frac{{N + d\left( {2 + N} \right)}}{1 - d}I_{o} $$

24 $$ I_{{L_{m} }} = I_{in} - I_{S} = I_{o} $$

As power switch *S* is turned on in mode 1, the peak current of this element can be obtained using average current as Eq. (25).

25 $$ i_{S\_peak} = \frac{{N + d\left( {2 + N} \right)}}{{d\left( {1 - d} \right)}}I_{o} $$

The peak current pathing from diodes are calculated as (26)- (28).

26 $$ i_{{D_{2} - peak}} = \frac{2 + N + Nd}{{1 - d}}I_{o} $$

27 $$ i_{{D_{1} - peak}} = i_{{D_{3} - peak}} = \frac{2}{1 - d}I_{o} $$

28 $$ i_{{D_{o} - peak}} = \frac{2}{d}I_{o} $$

Lastly, each component's root mean square (RMS) current is derived and presented. in (29)- (37).

29 $$ i_{S\_RMS} = \frac{{N + d\left( {2 + N} \right)}}{{\sqrt d \left( {1 - d} \right)}}I_{o} $$

30 $$ i_{{D_{1} - RMS}} = i_{{D_{3} - RMS}} = \frac{2}{{\sqrt {1 - d} }}I_{o} $$

31 $$ i_{{D_{2} - RMS}} = \frac{2 + N + Nd}{{\sqrt {1 - d} }}I_{o} $$

32 $$ i_{{D_{o} - RMS}} = \frac{2}{\sqrt d }I_{o} $$

33 $$ i_{{C_{1} - RMS}} = \left( {1 + N + d\left( {1 + N} \right)} \right)\sqrt {\frac{{1 + d - d^{2} }}{1 - d}} I_{o} $$

34 $$ i_{{C_{2} - RMS}} = \sqrt {\frac{{\left( {1 - d} \right)\left( {\left( {d + d^{2} } \right)\left( {1 + N} \right) - 2} \right)^{2} + d\left( {1 + N + d\left( {1 + N} \right)} \right)^{2} }}{{d\left( {1 - d} \right)}}} I_{o} $$

35 $$ i_{{C_{3} - RMS}} = i_{{C_{3} - RMS}} = i_{{N_{2} - RMS}} = \sqrt {\frac{1}{{d\left( {1 - d} \right)}}} \left( {2I_{o} } \right) $$

36 $$ i_{{N_{1} - RMS}} = \sqrt {\frac{{\left( {1 - d} \right)d\left( {1 + N + d\left( {1 + N} \right)} \right)^{2} + \left( {N + Nd + d - 1} \right)^{2} }}{1 - d}} I_{o} $$

37 $$ i_{{C_{o} - RMS}} = \sqrt {\frac{{d - d^{2} + \left( {2 - d} \right)^{2} }}{d}} I_{o} $$

## Design of the components

### Semiconductors

The power switch *S* must handle the peak current and voltage stresses without significant losses. Selection criteria include:

*Voltage Rating:* Should be higher than the peak input voltage.

*Current Rating:* Should handle the peak current during switch-on periods.

*Switching Speed:* Should be fast enough to minimize transition losses.

The diodes must have low forward voltage drop and fast recovery times to minimize losses and ensure efficient operation:

*Voltage Rating:* Higher than the maximum voltage across the diode.

*Current Rating:* Should handle peak current without significant heating.

### Inductors

The inductors play a critical role in energy storage and transfer:

Inductance Value for Lin: Should ensure Continuous Conduction Mode (CCM) operation under normal load conditions, reducing ripple and improving efficiency.

Coupled Inductor: The turns ratio *N* helps in adjusting voltage levels and improving power transfer capability. It should be designed to ensure efficient energy transfer. The minimum values of input inductor *L*_*in*_ and magnetizing inductance (*L*_*m*_) of the coupled inductors are obtained using (38) and (39).

38 $$ L_{in} = \frac{{dV_{{L_{in} }} }}{{f_{s} \Delta i_{{L_{in} }} }} \ge \frac{{dV_{{L_{in} }} }}{{2f_{s} I_{in} }} = \frac{{d^{2} V_{in} }}{{2f_{s} \left( {1 + N + d\left( {1 + N} \right)} \right)I_{o} }} $$

39 $$ L_{m} = \frac{{dV_{{L_{m} }} }}{{f_{s} \Delta i_{{L_{m} }} }} \ge \frac{{dV_{{L_{m} }} }}{{2f_{s} I_{{L_{m} }} }} = \frac{{d^{2} V_{in} }}{{2f_{s} \left( {1 - d} \right)I_{o} }} $$

In each operation mode, the leakage energy of the coupled inductor is managed as follows:

Mode 1: Energy is stored in the leakage inductance.

Mode 2: Leakage energy is transferred to capacitor *C*_*3*_ via diode *D*_*2*_, reducing energy losses.

Mode 3: Remaining leakage energy is transferred to the output, ensuring efficient energy utilization.

By appropriately switching the power components and selecting optimal values for the inductors and capacitors, the proposed DC-DC converter achieves high efficiency and reliable performance across all operation modes.

### Capacitors

Capacitors should have low Equivalent Series Resistance (ESR) to handle ripple currents efficiently:

Voltage Rating: Should be higher than the voltage across the capacitor. In this paper, assumption (40) is taken into account.

Current Rating: Should handle the ripple current without excessive heating. The equations for calculating the minimum values of capacitors *C*_*1*_- *C*_*o*_ are summarized in (41–45).

40 $$ \Delta V_{C} \le 2\% V_{C} $$

41 $$ C_{1} = \frac{{di_{{C_{1} }} }}{{f_{s} \Delta V_{{C_{1} }} }} \ge \frac{{di_{{C_{1} }} }}{{2\% f_{s} V_{{C_{1} }} }} = \frac{{\left( {1 + N + d\left( {1 + N} \right)} \right)I_{o} }}{{2\% f_{s} V_{in} }} $$

42 $$ C_{2} \ge \frac{{\left( {1 + N + d\left( {1 + N} \right)} \right)dI_{o} }}{{2\% f_{s} V_{in} }} $$

43 $$ C_{3} \ge \frac{{2I_{{_{o} }} }}{{2\% f_{s} \left( {1 + N} \right)V_{in} }} $$

44 $$ C_{4} \ge \frac{{2I_{{_{o} }} }}{{2\% f_{s} NV_{in} }} $$

45 $$ C_{o} \ge \frac{{\left( {1 - d} \right)dI_{o} }}{{2\% f_{s} \left( {1 + N + d\left( {1 + N} \right)} \right)V_{in} }} $$

## Experimental and comparison results

**Fig. 6:**
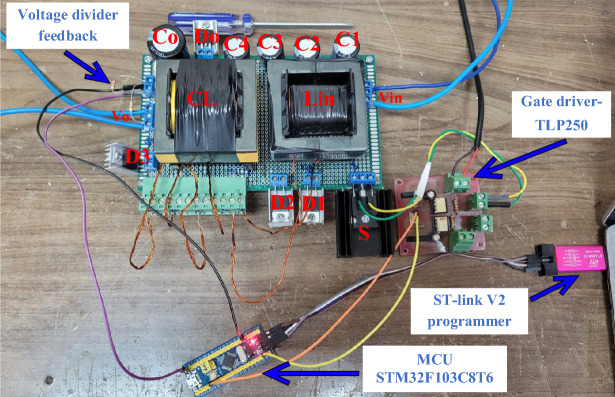
The Experimental Setup.

**Fig. 7:**
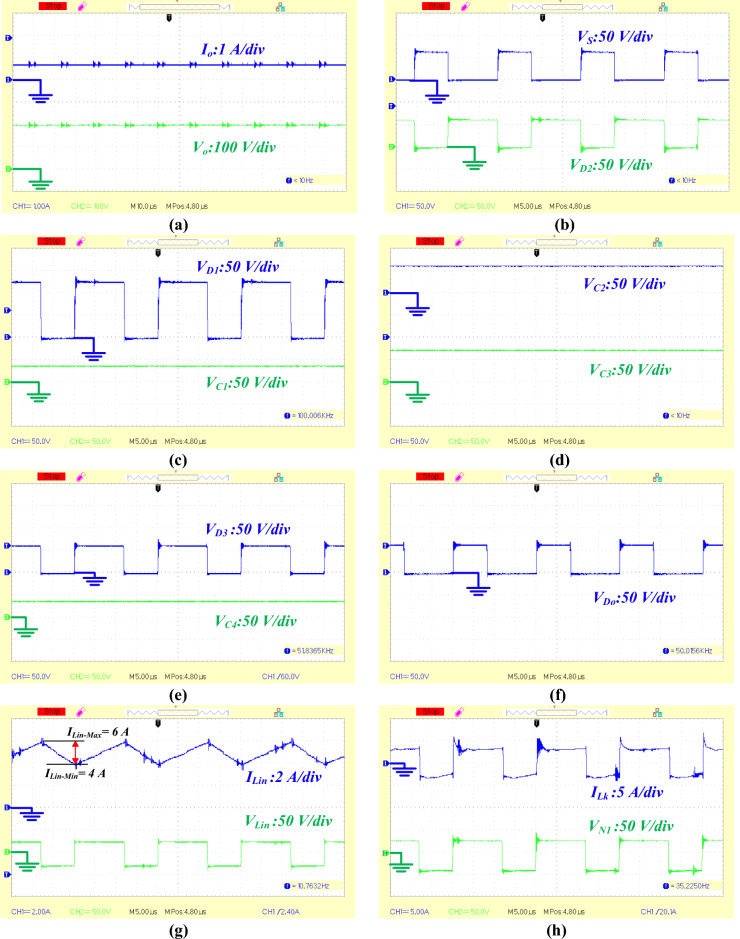
Experimental Waveforms.

**Fig. 8:**
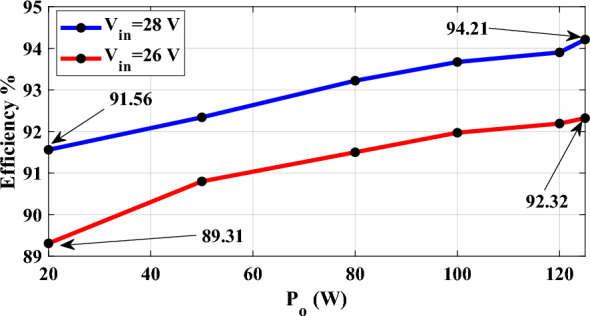
Efficiency versus output power.

This section describes the laboratory prototype's experimental results. To show the mathematical analysis, a 125 W system is designed with a switching frequency of 50 kHz and voltage conversion from 26 to 200 V. Table 2 shows the experimental characteristics and cost for each component. The experimental prototype is seen in Fig. 6. The experimental waveforms are shown in Fig. 7a–h. Based on these waveforms, the output voltage and current are 200 V and 0.625 A, respectively. As a result, the experimental voltage gain is 7.69, calculated as 200 V divided by 26 V. For *D* = 0.6 and *N* = 1, the theoretical voltage gain is 8. It is obvious that for *V*_*in*_ = 26 V, the theoretical output voltage is 208 V. Figure 7b depicts the voltage waveforms of power switch S and diode *D*_*2*_, where the maximum blocking voltage across power switch *S* and *V*_*D2*_ is measured 62 V. Figure 7c shows the voltage across diode *D*_*1*_ and capacitor *C*_*1*_, which are 124 V and 35 V, respectively. Figure 7d displays the voltages of capacitors *C*_*2*_ and *C*_*3*_, which are 62 V and 73 V, respectively. The voltage over diodes *D*_*3*_ and *D*_*o*_ is measured equal to 61 V and 62 V, respectively, while the measured voltage across capacitor *C*_*4*_ is 35 V. The maximum voltage stress over power switch *S* is equal to 62 V/200 V = 0.31. Furthermore, the maximum voltage stress of diodes is proportional to diode *D*_*1*_: 124 V/200 V = 0.62. The calculated voltage stress presented in Table 1 can be validated through these experimental results. Figures 7g, h illustrate the voltage and current waveforms of the input inductor and coupled inductor’s primary winding. Table 3 and Fig. 8 show the measured power efficiency of the proposed converter at various output power points and input voltages. The efficiency for *V*_*in*_ = 26 V and output power range of 20 W ~ 125 W was measured between 89.31 and 92.32%. Additionally, with *V*_*in*_ = 28 V, the efficiency ranges from 91.56 to 94.21%.

**Table 2:** Experimental characteristics.

Parameters		Values
*V*_*in*_	26 V	
*P*_*o*_	125 W	
*V*_*o*_	200 V	
*f*_*s*_	50 kHz	
Duty cycles	0.6	

Elements	Characteristic	
Switch (*S*)	*IFR260N* (price: 4.04 $)	
Diodes (*D*_*1*_ ~ *D*_*o*_)	*MUR1560* (price: 1.69 $)	
Capacitors	*C*_*1*_–*C*_*4*_	150 µF/ 200 V
		(price: 0.51 $)
		(aluminum electrolytic)
	*C*_*o*_	220 µF/ 250 V
		(price: 0.6 $)
		(aluminum electrolytic)
Inductor	*L*_*in*_ = 200 µH, *Ferrite core EE55/28/25*	
	(price: 5.17 $)	
Coupled inductor	*L*_*m*_ = 200 µH, *L*_*k*_ = 4 µH	
	*N*_*1*_*:N*_*2*_ = 1:1	
	*Ferrite core EE55/28/25*	
	(price: 5.17 $)

**Table 3:** Total loss in each power point.

Power (W)	*V*_*in*_ = 28 V		*V*_*in*_ = 26 V	
	Loss (W)	Efficiency %	Loss (W)	Efficiency %
20	1.84	91.56	2.39	89.31
50	4.14	92.34	5.06	90.8
80	5.81	93.22	7.43	91.5
100	6.75	93.67	8.73	91.97
120	7.79	93.9	10.16	92.19
125	7.68	94.21	10.39	92.32

**Fig. 9:**
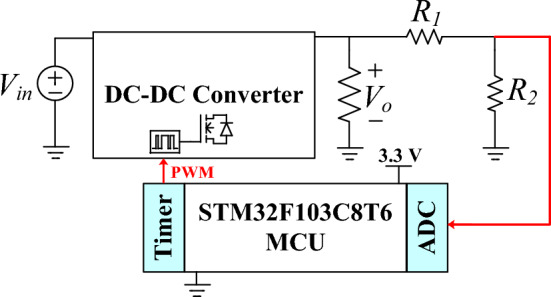
Closed-loop system.

To regulate the output voltage in response to load changes, a closed-loop system is introduced. The control method implemented in this work uses an STM32F103C8T6 ARM Cortex-M3 microcontroller to regulate the output voltage of the proposed converter as shown in Fig. 9. The following steps detail the process:

*1. Voltage Sensing and Conditioning:* To interface the high output voltage (200 V) of the converter with the microcontroller, a voltage divider circuit is employed. This circuit scales down the 200 V to a voltage level below 3.3 V, suitable for the used microcontroller's analog-to-digital converter (ADC). The voltage divider circuit is designed such that the maximum output voltage of 200 V is reduced to 3.3 V. This ensures that the input to the ADC remains within its operational range.

*2. Analog-to-Digital Conversion:* The STM32F103C8T6 microcontroller features 12-bit ADC units, which are configured to sample the conditioned voltage signal. Thus, the ADC converts the analog voltage ranging from 0 to 3.3 V) into a digital value between 0 and 4095 (2^12^–1 = 4095). This digital value represents the scaled version of the converter’s output voltage.

*3. Proportional-Integral (PI) Controller:* The digital value from the ADC is fed into a PI controller algorithm implemented as a function inside of the main codes. The PI controller calculates the error between the desired setpoint voltage and the measured output voltage. It then computes the necessary correction to minimize this error over time. The output of the PI function is used to adjust the duty cycle of a Pulse Width Modulation (PWM) signal.

**Fig. 10:**
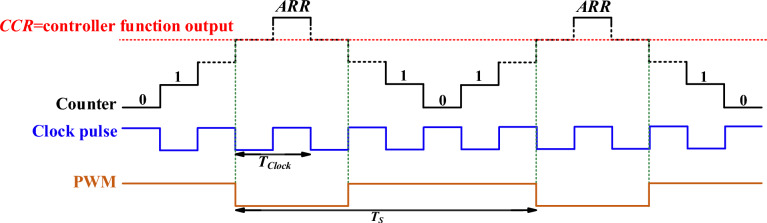
PWM generation in timer unit with center-aligned mode counting.

**Fig. 11:**
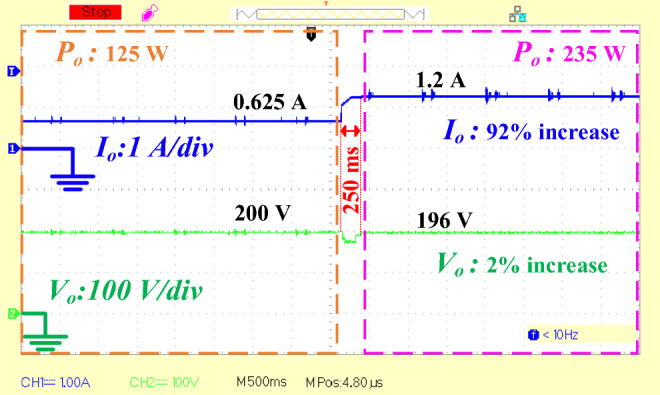
Closed -loop dynamic response of the output voltage.

*4. PWM Generation:* The computed duty cycle from the PI function is set to the capture compare register (CCR) of a timer unit within the microcontroller. One of the timer channels is configured to generate a PWM signal based on the duty cycle value. The clock frequency of the timer unit is set equal to 64 MHz The PWM frequency (50 kHz) is obtained using the following equation:

46 $$ f_{PWM} = \frac{{f_{clock} }}{(PSC + 1) \times 2(ARR + 1)} = \frac{64\,MHz}{{(0 + 1) \times 2(639 + 1)}} = 50\,kHz $$

where, *f*_*pwm*_ is the PWM frequency, *f*_*clock*_ is the clock frequency, *PSC* is the prescaler value, and *ARR* is the auto reload register (counter period) value. Figure 10 shows how PWM pulse is generated in the timer unit with center-aligned mode counting. This PWM signal controls the power switch of the converter, thereby regulating the output voltage. By integrating these components: voltage divider, ADC, PI controller function, and PWM generation, the system achieves stable control over the converter's output voltage. The experimental closed-loop dynamic response of the output voltage and current waveforms are shown in Fig. 11. Initially, the output voltage and current are set to 200 V and 0.625 A, respectively. When the output load suddenly increases from 125 W to 235 W, the output current rises from 0.625 A to 1.2 A. Despite this change, the output voltage is successfully regulated with only a 2% variation, and the transient response time is 250 ms. Finally, the proposed converter is compared with other similar converters in terms of component count, voltage gain, and voltage stress on semiconductors, as summarized in Table 4. Based on the theoretical and experimental results, it can be concluded that the proposed converter is a suitable choice for renewable energy systems, particularly photovoltaic energy systems, that require a high voltage DC bus with regulated voltage. Its high voltage gain, reduced voltage stress on semiconductor components, and efficient performance make it highly suitable for applications where stable and reliable voltage regulation is critical. The proposed converter's design enhances performance while simultaneously simplifying control and lowering costs, making it a vital part in the current renewable energy systems.

**Table 4:** Comparison with other works {R1-1&2, R2-2}.

	^2^	^17^	^28^	^12^	^16^	Proposed
Num. *S*	2	1	1	2	2	1
Num. *D*	2	3	3	5	5	4
Num. *C*	4	4	4	5	4	4
Num. *Core*	2	2	2	3	2	2
Num.*Windings*	3	3	3	6	4	3
*V*_*o*_*/V*_*in*_	$$\frac{N}{1 - d}$$	$$\frac{3N}{{1 - d}}$$	$$\frac{N + 2}{{1 - d}}$$	$$\frac{{\left( {8N + 2} \right) + 2d\left( {1 - 4N} \right)}}{1 - d}$$	$$\frac{3N + 1}{{1 - d}}$$	$$\frac{{1 + N + d\left( {1 + N} \right)}}{1 - d}$$
Max (*V*_*S*_*/V*_*o*_)	1/*N*	1/3*N*	$$\frac{1}{N + 2}$$	$$\frac{{3\left( {1 - d} \right)}}{{\left( {8N + 2} \right) + 2d\left( {1 - 4N} \right)}}$$	$$\frac{1}{3N + 1}$$	$$\frac{1}{{1 + N + d\left( {1 + N} \right)}}$$
Max (*V*_*D*_*/V*_*o*_)	1	$$\frac{3N - 1}{{3N}}$$	$$\frac{N + 1}{{N + 2}}$$	$$\frac{{N\left( {4 - 3d} \right)}}{{\left( {4N + 1} \right) + d\left( {1 - 4N} \right)}}$$	$$\frac{2N}{{3N + 1}}$$	$$\frac{1}{1 + d}$$
Cost $	24.2	21.85	21.85	35.04	29.27	23.78
Technique	VMC + CL	VMC + CL	VMC + CL	VMC + CL (Interleaved)	VMC + CL (Interleaved)	VMC + CL

## Conclusion

This paper has addressed the challenge of achieving high voltage gain with reduced voltage stress on semiconductor components in photovoltaic energy-based systems by presenting a novel converter design. The proposed converter utilizes a two-winding coupled inductor and voltage multiplier cells to boost the output voltage and serve as voltage clamps for the power switch, thereby enhancing performance. The single switch design simplifies control, reduces costs, and improves reliability. The converter boasts key advantages such as a low component count, a common ground between input and output ports, and high efficiency. A thorough investigation of the converter's function was conducted through mode analysis and steady-state analysis. A 125 W prototype operating at a 50 kHz switching frequency was built to validate the theoretical analysis. In the experimental results, a closed-loop system was presented for the proposed converter, further demonstrating its effectiveness. The experimental results confirmed the practicality of the high voltage gain converter, with a maximum efficiency of 94.21% at the rated power of 125 W.

## Data Availability

The data that support the findings of this study are available from the corresponding author upon reasonable request.
